# Composite multiscale chemical informatics for formulations: challenges and solutions

**DOI:** 10.1186/1758-2946-3-S1-O13

**Published:** 2011-04-19

**Authors:** Johannes GEM Fraaije, Ruben Serral Gracia

**Affiliations:** 1 Leiden University, The Netherlands; 2 Culgi BV, The Netherlands

## 

Chemical Informatics lives in a twilight zone between informatics and mathematics on the one hand, and physical chemistry on the other hand. One could count numerous success stories of application in chemical and pharmaceutical industries. So far, the focus has been on correlating some molecular descriptor with measured activity. New challenges arise when one wishes to extend application into such complex problems as steered self-assembly, or, as it would be known in industry, formulation. One may think of, for example, correlating complex polymer and surfactant mixtures for some drug delivery system. But applications also include finding better ways to stabilize asphaltene aggregates in oil recovery; or finding more efficient ways to design laundry softeners; or to design high impact composite materials. In all these areas, and still more, taken from real cases studies in industrial soft matter research, one is interested in understanding and designing some aggregation on length and time scales that surpass traditional molecular modeling. Such self-assembly process can be understood by applying concepts from multiscale modeling, that combine techniques from quantum to atomistic to coarse grained modeling. Multiscale modeling is finding rapid inroad into industrial research as a pragmatic way to address problems that could not be addressed at all by traditional molecular modeling. The question then naturally arises, if one could combine chemical informatics with multiscale modeling, in the spirit of past successes of Chemical Informatics in combination with molecular modeling, in such way to extend the application even further.

I will discuss these novel concepts in the framework of the Chemistry Unified language Interface (CULGI), a generic scriptable computational platform for multiscale modeling, including statistical analysis. CULGI is developed in the context of a large international industrial consortium, with offices in Europe, USA and China. Sponsors include several government EU projects NANOMODEL and SELFMEM, and Dutch Aptalife. The CULGI software is distributed through an extensive service network.

